# Supplementary material for: Mobile phone MIMO antenna array miniaturization-based low SAR research in the combined EMF
Source: PLoS One. 2026 Jan 14;21(1):e0340681. doi: 10.1371/journal.pone.0340681 (PMC12803447; doi:10.1371/journal.pone.0340681)
Supplement: Attachment — Submitted filename: Response to reviewers.docx [file pone.0340681.s001.docx]

Dear Editors and Reviewers:

Thank you for your response and comments concerning our manuscript entitled “*Mobile phone MIMO antenna array miniaturization-based low SAR research in the compound microwave electromagnetic field*” (Manuscript reference: PONE-D-25-40499, original title: “*Mobile phone MIMO antenna array miniaturization-based low SAR research in the compound microwave electromagnetic field*”). All the comments are valuable and very helpful for revising and improving our paper. We hope that our response and corrections can meet your expectations. Besides that, the English language of our manuscript has been further improved based on the editor's comments, and the revised part are marked in red.

The responses to editor's comments are as follows. Responses to the two reviewers are listed in the two tables below.

**Editor comment 1:**

Please ensure that your manuscript meets PLOS ONE's style requirements, including those for file naming. The PLOS ONE style templates can be found at

https://journals.plos.org/plosone/s/file?id=wjVg/PLOSOne_formatting_sample_main_body.pdf and

https://journals.plos.org/plosone/s/file?id=ba62/PLOSOne_formatting_sample_title_authors_affiliations.pdf.

**Answer:**

According to comment 1, we revise the manuscript as requested.

**Editor comment 2:**

Please note that PLOS One has specific guidelines on code sharing for submissions in which author-generated code underpins the findings in the manuscript. In these cases, we expect all author-generated code to be made available without restrictions upon publication of the work. Please review our guidelines at https://journals.plos.org/plosone/s/materials-and-software-sharing#loc-sharing-code and ensure that your code is shared in a way that follows best practice and facilitates reproducibility and reuse.

**Answer:**

According to comment 2, we have no code in this article, but the data we need in this article can be provided without restriction.

**Editor comment 3:**

Thank you for stating in your Funding Statement:

“This work was supported by the National Natural Science Foundation of China: [grant number 62161017, 61701208，52467026]; The Department of Education of Gansu Province: [grant number 2024CXPT-11]; The Joint Research Foundation of Gansu Province: [grant number 24JRRA858].”

Please provide an amended statement that declares *all* the funding or sources of support (whether external or internal to your organization) received during this study, as detailed online in our guide for authors at http://journals.plos.org/plosone/s/submit-now. Please also include the statement “There was no additional external funding received for this study.” in your updated Funding Statement.

**Answer:**

According to comment 3, we have revised the funding statement, as follows:

This work was supported by the National Natural Science Foundation of China: [grant number 62161017, 61701208，52467026]; The Department of Education of Gansu Province: [grant number 2024CXPT-11]; The Joint Research Foundation of Gansu Province: [grant number 24JRRA858]; Zhiyuan Laboratory: [grant number ZYL2024004]. There was no additional external funding received for this study. The funders had no role in study design, data collection and analysis, decision to publish, or preparation of the manuscript.

**Editor comment 4:**

Thank you for stating the following financial disclosure:

“This work was supported by the National Natural Science Foundation of China: [grant number 62161017, 61701208，52467026]; The Department of Education of Gansu Province: [grant number 2024CXPT-11]; The Joint Research Foundation of Gansu Province: [grant number 24JRRA858].”

Please state what role the funders took in the study. If the funders had no role, please state: "The funders had no role in study design, data collection and analysis, decision to publish, or preparation of the manuscript."

If this statement is not correct you must amend it as needed.

Please include this amended Role of Funder statement in your cover letter; we will change the online submission form on your behalf.

Please include your amended Funding Statement within your cover letter. We will change the online submission form on your behalf.

**Answer:**

According to comment 4, we have revised the funding statement in the cover letter.

The funders had no role in study design, data collection and analysis, decision to publish, or preparation of the manuscript.

**Editor comment 5:**

We note that your Data Availability Statement is currently as follows: [All relevant data are within the manuscript and its Supporting Information files.]

Please confirm at this time whether or not your submission contains all raw data required to replicate the results of your study. Authors must share the “minimal data set” for their submission. PLOS defines the minimal data set to consist of the data required to replicate all study findings reported in the article, as well as related metadata and methods (https://journals.plos.org/plosone/s/data-availability#loc-minimal-data-set-definition).

For example, authors should submit the following data:

- The values behind the means, standard deviations and other measures reported;

- The values used to build graphs;

- The points extracted from images for analysis.

Authors do not need to submit their entire data set if only a portion of the data was used in the reported study.

If your submission does not contain these data, please either upload them as Supporting Information files or deposit them to a stable, public repository and provide us with the relevant URLs, DOIs, or accession numbers. For a list of recommended repositories, please see https://journals.plos.org/plosone/s/recommended-repositories.

If there are ethical or legal restrictions on sharing a de-identified data set, please explain them in detail (e.g., data contain potentially sensitive information, data are owned by a third-party organization, etc.) and who has imposed them (e.g., an ethics committee). Please also provide contact information for a data access committee, ethics committee, or other institutional body to which data requests may be sent. If data are owned by a third party, please indicate how others may request data access.

**Answer:**

The data availability statement is correct. All relevant data are within the manuscript and its Supporting Information files.

**Editor comment 6:**

If the reviewer comments include a recommendation to cite specific previously published works, please review and evaluate these publications to determine whether they are relevant and should be cited. There is no requirement to cite these works unless the editor has indicated otherwise.

**Answer:**

Since the reviewers did not recommend citing specific previously published works, we have not added any new citations. We have revised the citation format and descriptions of the references according to the reviewers' suggestions.

**Table 1 of the modifications according to reviewer 1**

| **Comments from the Reviewer 1** | **Modifications** |
| --- | --- |
| 1.The overall structure is logical, but there are several grammatical and syntactical issues that should be corrected for clarity. | According to comment 1, we have corrected the grammatical and syntactical issues in the manuscript. Please refer to the annotations section in the revised manuscript. |
| 2. The SAR distribution figures are informative, but the resolution could be improved. It would also help to include a summary table that clearly compares SAR values across different scenarios (single 4G, single 5G, and compound exposure), and across different body tissues. | According to comment 2, we updated the figures with higher resolution. The summary table according to your suggestion is added to Section 4, as follows:  “Then, we compare the multi-frequency exposure results with the single-frequency exposure results, with the maximum values shown in Table 6 and the distributional differences shown in Figs 6-12.    ” |
| 3.The use of multi-pose human models adds robustness and realism to the simulation setup. However, the paper would benefit from additional clarification on the following:  (a)How were the poses selected, and do they represent statistically common user behaviors?  (b)Were the MIMO antenna configurations consistent across the 4G and 5G bands, and how were mutual coupling effects between antennas treated in the simulation?  (c)Was the SAR averaged over standard mass values (1g or 10g), and were peak spatial-average SAR values reported in accordance with international guidelines (e.g., ICNIRP, IEEE)? | According to comment 3, the explanations are as follows.   (a) The poses were selected based on common daily smartphone usage scenarios and represent statistically common user behaviors. During a phone call, the phone is typically held to the side of the head. When operating (typing, texting), using one hand (portrait) or two hands (landscape) to hold the phone is both common postures, which have been documented in studies across various fields.   As noted in the study “*The relationship between smartphone usage position, pain, smartphone addiction, and hand function*” (DOI: 10.3233/BMR-240154), “Generally, the typical smartphone posture involves holding the device below eye level with one or both hands, neck flexion to look at the device, elbow flexion, and wrist extension to keep the phone, and using the thumb to touch the screen.”  Furthermore, the postures illustrated in Fig. 2 of “*Damaged/missing proximity sensor induces screen mistouch when answering calls: Prediction of smartphone answering status by posture data*” (DOI: 10.3233/BMR-240154) and Figure 1 of “*Investigating the Effects of Encumbrance on One- and Two-Handed Interactions with Mobile Devices*” (DOI: 10.1145/2556288.2557312) are consistent with the posture in Fig. 4 of our study.        Statistical findings from the study “*Mobile Phone Use Behaviors and Postures on Public Transportation Systems*” (DOI: 10.1371/journal.pone.0148419) show that “Overall, the different-hand group made up the highest proportion (40%), closely followed by the same-hand group (30%) and then by the both-hands group (22%).” Moreover, “The standing users had a higher proportion of using the same hand to hold/operate,” “It was observed that 46% of standing passengers used the same hand to hold/operate the phones (same-hand group).”  Collectively, findings from the above studies validate the statistical representativeness of the poses selected in our research.   (b) The MIMO antenna configurations were not consistent across the 4G and 5G bands; they were differentiated by antenna types and covered frequency ranges. These two issues are explained in Section 2, Ant1 and Ant2 mainly cover the 2G and 5G bands. Ant3, Ant4, Ant5 and Ant6 mainly cover the 3G and 4G bands. And the S-parameters are detailed in Fig. 2. In order to reduce the coupling effect between the array elements during the miniaturization of this antenna array, the decoupling unit consisting of a metamaterial unit and a waveguide is added.     **Fig 2. S-parameter of the miniaturized mobile phone MIMO antenna array.**  “As shown in Fig 2a, the comparison results show that the coefficients of Ant 1 and Ant 2 (*S*_11_, *S*_22_) below -6 dB cover the range of 1.86 GHz ~ 1.92 GHz and 3.41 GHz ~ 3.58 GHz, which satisfy the 2G and 5G requirements.”  “As shown in Fig 2c, the measurement bandwidths of the reflection coefficients of Ant 4 and Ant 5 (*S*_44,_ *S*_55_) below -6 dB are 2.55 GHz ~ 2.61 GHz and 2.55 GHz ~ 2.61 GHz, satisfying the communication requirements of the 4G frequency band (2.59 GHz-2.66 GHz). Fig 2d-f shows the coupling coefficients (*S*_21_, *S*_31_, *S*_53_, *S*_63_, *S*_43_, *S*_56_) between pairs of radiating units, and their measured results are below -15 dB, which meet the design requirements.”   (c) The licensed version of COMSOL Multiphysics 5.2 was purchased by our research team to simulate SAR. Notably, this software version is only capable of computing peak local SAR values. In terms of the computational electromagnetic dosimetry, if the calculated peak local SAR values do not exceed the regulatory limit, the average SAR values would also be below the limit. Consequently, comparisons between the peak local SAR values and the regulatory limit of ICNIRP have been incorporated into Section 3.2 of the manuscript, as follows:   “The above results show the radiation impact when users operate a single function of mobile phone MIMO antenna array, with all peak SAR values under single-frequency exposure remaining below the ICNIRP safety limit of 2 W/kg.” |
| 4.While the paper successfully demonstrates the increased SAR due to compound exposure, it would be valuable to contextualize these findings within the framework of current EMF safety standards. For instance:  (a)Do the maximum SAR values in the compound scenario exceed regulatory limits?  (b)How does the reduction in SAR from the miniaturized MIMO antenna compare quantitatively to conventional antenna designs? | (a) The maximum SAR value obtained in the compound exposure scenario is 0.343 W/kg, which does not exceed the limit of ICNIRP guidelines of 2 W/kg. And as suggested, we have incorporated a comparison table in Section 4 of the revised manuscript, as follows:   “Then, we compare the multi-frequency exposure results with the single-frequency exposure results, with the maximum values shown in Table 6 and the distributional differences shown in Figs 6-12.    ”   (b) Regarding the quantitative comparison of SAR reduction between the miniaturized MIMO antenna and conventional antenna designs, it should be clarified that the present study focuses on the influence of user postures on SAR distribution and SAR absorption in different biological tissues. Due to space constraints of the manuscript, a dedicated quantitative analysis of SAR reduction between the two antenna types has not been included in this work.   The quantitative comparison of SAR reduction is provided in our previous study, “*A novel radiation protection method for miniaturized MIMO mobile terminal antenna design based on metamaterials*” (DOI: 10.1371/journal.pone.0323299). The findings demonstrate that the miniaturized antenna significantly reduces SAR in head tissues compared to the original conventional design. Furthermore, the miniaturized antenna can effectively alleviate the trend of SAR increasing with input power. The specific values are shown in the table below (https://doi.org/10.1371/journal.pone.0323299.t003).   |

**Table 2 of the modifications according to reviewer 2**

| **Comments from the Reviewer 2** | **Modifications** |
| --- | --- |
| The term “compound electromagnetic field” is not standard in EMF research. Please replace it with a more conventional term. | As suggested, we have replaced the non-standard term “compound electromagnetic field” with the more conventional terms “combined EMF exposure” and “multi-frequency exposure”, both of which are used in the ICNIRP guidelines. These terms are consistent with that employed in recent publications, such as “*Assessment of combined exposure to intermediate-frequency electromagnetic fields and pulsed electromagnetic fields among library workers in Japan*” (DOI: 10.3389/fpubh.2022.870784) and “*Simultaneous 4G and 5G EMF Exposure and Field Uniformity in a Reverberation Chamber for Animal Studies*” (DOI: 10.3390/app151810286). |
| Title can be improved for clarity and conciseness. | Thank you for your suggestion, we have changed the title to “*Mobile phone MIMO antenna array miniaturization-based low SAR research in the combined EMF*”. |
| The manuscript contains numerous grammatical and technical issues. The entire paper should undergo professional English editing by a native scientific editor. | Thank you for your suggestion, we have revised and polished the manuscript accordingly. Please refer to the annotations section in the revised manuscript. |
| Please improve figure readability and ensure sequential referencing in text. | Thank you for your suggestion, we have revised the figures accordingly. |
| Most citations are recent and relevant but not consistently formatted. | Thank you for your suggestion. We have revised the citation format to ensure consistency. |
| The paper builds on the authors’ previous PLOS ONE work (Ref. [20]) but does not clearly distinguish new contributions. Authors should emphasize what is new in this manuscript. | Our previous research proposed a compact cellphone antenna based on metamaterials and discussed the impact of antenna miniaturization on the human head tissues at a single frequency. To further evaluate the proposed miniaturized antenna effect on the whole body with multi-pose, this study analyzed the SAR radiated by different vital tissues (brain, thyroid, heart, lungs, and liver), and compared the differences between single-frequency and multi-frequency exposure effects.  In response to your comment, the revised manuscript now clarifies the new contributions of this study relative to our previous work (Ref. [20]) in Section 3.1, as follows:  “Our previous research primarily focused on exploring an innovative approach to reduce radiation dose absorption within human head tissues [20]. In order to systematically analyze the near-field electromagnetic exposure effect of mobile phone MIMO antenna array on the human body, we construct electromagnetic simulation models for multi-pose, including calling pose, one-handed operation pose and two-handed operation pose, as shown in Fig 4.” |
| The simulation lacks key details necessary for reproducibility. Author is suggested to add a detailed subsection on simulation details and validation. | Thank you for your suggestion. This study employs COMSOL Multiphysics for RF electromagnetic wave simulation, with the core solution workflow following Fig 1.    Fig 1. COMSOL simulation workflow diagram.  Taking the two-handed operation posture as an example, the automatic meshing method was adopted, allowing the mesh generator to select the optimal algorithm for each domain. The model was discretized into a total of 1,205,561 elements. The MIMO antenna array was excited by lumped ports, and an input power of 0.05 W. A cuboid scattering boundary condition was established outside the human body model, and calculations were performed using a direct solver. The mesh division and COMSOL simulation workflow are illustrated in Fig 2.    Fig 2. Mesh plot. |
| Author did not provide any experimental validation. Suitable benchmark SAR studies or validation of model against any standard systems should be included. Author should also include uncertainty or sensitivity analysis. | Thank you for your suggestion. In Section 2 of the manuscript, we have verified the reliability of the exposure source through actual measurements of the fabricated MIMO antenna prototype. It should be noted that experimental verification of SAR in humans faces ethical constraints and technical challenges. In electromagnetic exposure research, simulation based on computational electromagnetics, epidemiological investigation, and experimental investigation of plants and animals are all recognized as valid research methods. Among these, calculating human SAR using simulation software such as COMSOL has become a reliable and widely adopted approach in this field.  This study aims to evaluate the radiation dose absorbed by diverse human tissues with different poses, and verify the electromagnetic exposure safety of the miniaturized MIMO terminal antenna array. The uncertainty or sensitivity analysis would be our next work, which focuses on the mobile terminal design. |
| Methodological description lacks sufficient detail for full reproducibility. | Thank you for your suggestion. The key details of the simulation are as follows:  For a single operating frequency, this paper follows the standard definition of local SAR as per IEEE C95.1-2019:  $SAR=\frac{\sigma E^{2}}{2\rho}$  Independent calculations are performed for each frequency to obtain the single-frequency SAR distribution. This definition is also adopted by the ICNIRP (2020) radiofrequency exposure guidelines.  Secondly, according to the stipulations in ICNIRP (2020) regarding the superposition of multi-source/multi-frequency radiofrequency exposures, for the same mass-averaged conditions (including local SAR and whole-body average SAR), the SAR values at different frequencies can be calculated separately and evaluated in a superimposed manner. The superposition formula provided by ICNIRP is as follows:  $\sum_{i=100GHz}^{300GHz} \frac{{SAR}_{i}}{{SAR}_{BR}}$ ≤1  This formula indicates that SAR contributions from different frequencies are additive under the same mass-averaged condition.  In our article, we first cite the calculation formula of ${SAR}_{combind}$ in Ref. [30], and then explain the combined electric field strength $\left\vert\overset{̑}{E} \right\vert$ is the sum of the electric field intensities corresponding to different frequencies in the x, y, and z components, as Equation 2. Finally, we propose our advanced SAR_combind_ calculation method by taking into account the permittivity and conductivity of every human tissue in different operation frequencies, as Equation 3.  In COMSOL, independent study steps can be established for each frequency to compute the corresponding SAR. The combined SAR can be directly obtained during post-processing using the expression “withsol('sol1', emw.SAR) + withsol('sol2', emw.SAR)”, thereby yielding the local SAR under combined electromagnetic exposure. It should be noted that, during the combined calculation, the permittivity of the human tissues corresponding to each single frequency is all set in the software. |
| Statements such as “penetration of electromagnetic waves is enhanced under compound EMF exposure” are not physically proved. It is suggested to please revise the discussion to provide consistent explanation. | Thank you for your suggestions, we have revised the relevant expressions in Section 4 of the manuscript accordingly, as follows:  “The radiation doses under combined EMF exposure are several times higher than those under single-frequency EMF exposure, indicating that such exposure scenarios may lead to a significant increase in radiated energy absorption by various human tissues.” |
| Figures 6–12 lack clear scale bars and detailed legends. Author is suggested to add scale/color bars showing SAR values and label anatomical regions. | Thank you for your suggestions, we have revised Figs 6-12 as suggested. The revised figures are as follows:    **Fig 6. Comparison of whole-body SAR distributions between single and combined EMF exposure (Unit: W/kg).** (a) Calling pose. (b) One-handed operation pose. (c) Two-handed operation pose.    **Fig 7. Comparison of skull tissue SAR distributions between single and combined EMF exposure (Unit: W/kg).** (a) Calling pose. (b) One-handed operation pose. (c) Two-handed operation pose.    **Fig 8. Comparison of brain tissue SAR distributions between single and combined EMF exposure (Unit: W/kg).** (a) Calling pose. (b) One-handed operation pose. (c) Two-handed operation pose.    **Fig 9. Comparison of thyroid tissue SAR distributions between single and combined EMF exposure (Unit: W/kg).** (a) Calling pose. (b) One-handed operation pose. (c) Two-handed operation pose.    **Fig** **10. Comparison of heart tissue SAR distributions between single and combined EMF exposure (Unit: W/kg).** (a) Calling pose. (b) One-handed operation pose. (c) Two-handed operation pose.    **Fig** **11. Comparison of lung tissue SAR distributions between single and combined EMF exposure (Unit: W/kg).** (a) Calling pose. (b) One-handed operation pose. (c) Two-handed operation pose.    **Fig** **12. Comparison of liver tissue SAR distributions between single and combined EMF exposure (Unit: W/kg).** (a) Calling pose. (b) One-handed operation pose. (c) Two-handed operation pose.  In this study, the SAR distribution is presented and analyzed based on tissue types rather than anatomical regions. This approach is standard in computational human body electromagnetic exposure research, as the dielectric properties—which are the critical parameters determining RF energy absorption—are defined by biological tissue type (e.g., muscle, fat, bone) rather than by anatomical region. |
| The discussion of Table 5 is minimal. An explanation on why miniaturization reduces SAR would be a helpful addition for the readers. | Thank you for your suggestions. We have revised the discussion of Table 5, as follows:  “Specifically, Table 5 illustrates that under near-field exposure scenarios, the higher the operating frequency of the antenna, the greater the radiated EMF energy, which in turn leads to a higher SAR in tissues close to the exposure source. When antennas operate at the same frequency, a more compact antenna size corresponds to less radiated energy, resulting in a lower SAR in tissues close to the exposure source. Compared to other designs in the literature, the proposed antenna exhibits advantages of a lower profile, more compact size, and minimal radiation impact on human tissues.” |
| Some references (e.g., [12]–[14]) need context (which safety standards are meant — IEEE, etc.?). | Thank you for your suggestions. We have revised the relevant expressions in Section 1 of the manuscript accordingly. The revised part is as follows:  “Studies based on electromagnetic simulations indicate that the radiation levels of smartphones in typical usage scenarios are usually below the exposure limit of 2 W/kg set by the International Commission on Non-Ionizing Radiation Protection (ICNIRP) guideline [12]. However, SAR may exceed the safety limits when the exposure source is too close to the body [13,14].” |
| The inclusion of seven tissues (scalp, skull, brain, thyroid, heart, lungs, liver) is adequate for preliminary SAR analysis, but the model excludes high-exposure regions such as skin, ear, and hand. | Thank you for your suggestion. In our previous research study (Ref. [20], titled “*A novel radiation protection method for miniaturized MIMO mobile terminal antenna design based on metamaterials*”, DOI: 10.1371/journal.pone.0323299), we have already analyzed the specific electromagnetic exposure dose distribution in the head.  The focus of this paper is on the electromagnetic exposure of key organ tissues within the whole-body human model, as well as a comparative analysis of the electromagnetic exposure doses under single frequency and multi-frequency electromagnetic fields. Therefore, this paper primarily discusses the electromagnetic exposure across the entire body. |
| Please ensure that units (S/m for conductivity, dimensionless for permittivity) are stated in Table 1. | Thank you for your suggestion, we have listed the required units in Table 1 as shown below:   |
| Conclusion must be improved in term of language and clearer phrasing. Some sentences are grammatically incorrect or ambiguous (e.g., “the organ has affected a lot”), and certain claims such as “penetrability decreases” or “SAR distribution areas expand significantly” should be rephrased more precisely. The authors should also replace wording like “a lot” and “obvious difference.” With scientific wording. Moreoever, SAR increase under compound EMF exposure is not necessarily due to deeper penetration. | Thank you for your suggestion, we have revised the manuscript accordingly. The conclusion is as follows:  “In this study, we established different scenarios of public mobile phone usage, and explored an active electromagnetic exposure protection approach based on miniaturized radiation source design. Through simulating electromagnetic exposure of a human model in multi-pose, we analyzed the maximum radiation dose absorbed at different frequencies. The findings indicate that during the calling posture the peak SAR values are observed in the head tissues and thyroid tissue. Conversely, in the one-handed and two-handed operation postures, the peak SAR values are present in the thyroid tissue and trunk tissues. As frequency increases, the SAR values in external tissues show a more significant increase compared to those in internal tissues of the human body. The peak SAR values in diverse tissues under combined EMF exposure are 1.02 to 15.85 times higher than those under single EMF exposure. Moreover, in comparison to single EMF exposure, the SAR distributions across diverse tissues demonstrate an overall higher dose level under combined EMF exposure. The uncertainty or sensitivity analysis would be our next work.” |
| It is suggested to rephrase the paragraph of conclusion to form a coherent summary instead of bullet-style listing. | Thank you for your suggestion, we have revised the manuscript accordingly. The conclusion is as follows:  “In this study, we established different scenarios of public mobile phone usage, and explored an active electromagnetic exposure protection approach based on miniaturized radiation source design. Through simulating electromagnetic exposure of a human model in multi-pose, we analyzed the maximum radiation dose absorbed at different frequencies. The findings indicate that during the calling posture the peak SAR values are observed in the head tissues and thyroid tissue. Conversely, in the one-handed and two-handed operation postures, the peak SAR values are present in the thyroid tissue and trunk tissues. As frequency increases, the SAR values in external tissues show a more significant increase compared to those in internal tissues of the human body. The peak SAR values in diverse tissues under combined EMF exposure are 1.02 to 15.85 times higher than those under single EMF exposure. Moreover, in comparison to single EMF exposure, the SAR distributions across diverse tissues demonstrate an overall higher dose level under combined EMF exposure. The uncertainty or sensitivity analysis would be our next work.” |
